# A Minimally Invasive Approach Towards “Ecosystem Hacking” With Honeybees

**DOI:** 10.3389/frobt.2022.791921

**Published:** 2022-04-28

**Authors:** Martin Stefanec, Daniel N. Hofstadler, Tomáš Krajník, Ali Emre Turgut, Hande Alemdar, Barry Lennox, Erol Şahin, Farshad Arvin, Thomas Schmickl

**Affiliations:** ^1^ Artificial Life Lab, Institute of Biology, University of Graz, Graz, Austria; ^2^ Artificial Intelligence Centre, Faculty of Electrical Engineering, Czech Technical University, Prague, Czechia; ^3^ Department of Mechanical Engineering, Middle East Technical University, Ankara, Türkiye; ^4^ ROMER-Center for Robotics and Artificial Intelligence, Middle East Technical University, Ankara, Türkiye; ^5^ Department of Computer Engineering, Middle East Technical University, Ankara, Türkiye

**Keywords:** honeybees, micro-robotics, ecosystem hacking, swarm robotics, queen behavior

## Abstract

Honey bees live in colonies of thousands of individuals, that not only need to collaborate with each other but also to interact intensively with their ecosystem. A small group of robots operating in a honey bee colony and interacting with the queen bee, a central colony element, has the potential to change the collective behavior of the entire colony and thus also improve its interaction with the surrounding ecosystem. Such a system can be used to study and understand many elements of bee behavior within hives that have not been adequately researched. We discuss here the applicability of this technology for ecosystem protection: A novel paradigm of a minimally invasive form of conservation through “Ecosystem Hacking”. We discuss the necessary requirements for such technology and show experimental data on the dynamics of the natural queen’s court, initial designs of biomimetic robotic surrogates of court bees, and a multi-agent model of the queen bee court system. Our model is intended to serve as an AI-enhanceable coordination software for future robotic court bee surrogates and as a hardware controller for generating nature-like behavior patterns for such a robotic ensemble. It is the first step towards a team of robots working in a bio-compatible way to study honey bees and to increase their pollination performance, thus achieving a stabilizing effect at the ecosystem level.

## 1 Introduction

The queens of (eu)social insect colonies are arguably the most influential organisms within their surrounding ecosystems: They produce thousands, sometimes millions, of workers that spread out from their nests into the surrounding environment to forage and harvest, affecting ultimately billions of other organisms each day (Brian, 2013). The ecological role of these social superorganisms is present at all trophic levels, such as ants acting as dispersers of seeds of primary producers (Detrain and Tasse, 2000). Social insects are acting as predators (Way and Khoo, 1992), and as facilitators in the decomposition of organic materials (McGlynn and Poirson, 2012; Eubanks et al., 2019), similar to the role of termite colonies (Poulsen et al., 2014). Sometimes, social insects also get the short end of the stick and end up as prey (Pascual-Garrido et al., 2013). Eusocial wasps do not only regulate the ecological stability due to their predation activity (Prezoto et al., 2019), as they can also be pollinators (Mello et al., 2011).

However, the most influential superorganisms in the world are honeybee colonies, where tens of thousands of workers gather food. The pollination of higher plants in an area of up to 280 km^2^ around a single beehive, calculated according to (Beekman and Ratnieks, 2000), represents a significant ecosystem service, with activity levels of 20,000 or more pollination flights per colony per day, assuming a volume of 10,000 foraging bees with 20 flights on average per day. Such an intensive and wide-spread pollination service towards plants significantly supports vegetational spreading and plants’ reproduction, ultimately yielding support of food supply for higher trophic levels, i.e., animals. In addition to these fairly obvious effects of pollination services, support for higher plants has microclimatic as well as landscape effects, as plants provide building and nesting materials, as well as shelters, *e.g.*, for protection from predators.

An important aspect of honeybee colony organization is the degree of centralism built on the queen: All honeybee foragers in the colony, and thus all ecosystem-active work of the colony, originate from a single queen. This queen exerts her influence on the worker bees in various ways and is thus a central element within the otherwise mostly decentralized self-regulating system of a honeybee colony (Seeley, 2009).

In addition to purely scientific curiosity, the study of these animals is important for understanding the colony system for two other reasons: First, ecosystems are under severe stress today (Hallmann et al., 2017) with eusocial insects being a keystone species (Boswell et al., 1998). Thus they are essential for their ecosystem’s stability and even beyond the range of their ecosystem (Easton-Calabria et al., 2019). On the one hand, they are affected by climate change (Le Conte and Navajas, 2008; Menzel and Feldmeyer, 2021). In honeybees, anthropogenic stresses can lead to the phenomenon of Colony Collapse Disorder (Williams et al., 2010) and eventually the death of a colony. On the other hand, climate change can favor invasive species spreading, and therein the spreading of invasive superorganisms is an especially significant threat to ecosystems (Bertelsmeier, 2021). This threat spectrum ranges from Africanized bees to invasive hornet species to fire ants, which occasionally get major media attention for a news cycle, but these cases represent only the tip of the iceberg.

Second, social insect colonies are of high socio-economic value, *e.g.,* wasp colonies or ant colonies can be important factors in securing agriculture by predating unfavorable organisms such as parasites or pests (Way and Khoo, 1992; Prezoto et al., 2019). The socio-economic value of honeybees in food production by pollinating fruiting plants is of enormous significance. Insect pollination, mostly by bees, is necessary for 75% of all crops that are used directly for human food worldwide (Klein et al., 2007). The value of pollination services provided by insect pollinators, especially bees, is estimated at 153 billion euros in 2005 for the main crops that feed the world (Gallai et al., 2009).

Here we explore the role that bioinspired and biomimetic robots can play in mitigating these problems, as well as their role as an investigative tool for behavioral rules within a superorganism. For a successful integration, however, it is essential that the robots are accepted by the target colony. To achieve this, many physical parameters of the robot must be precisely tuned (smell, taste, texture, *etc.*), but also the behavior of the artificial agent must be accepted by the natural animals.

Biomimetic robots have been associated with animals that live in groups (Halloy et al., 2007; Bonnet et al., 2018) or even eusocially together (Landgraf et al., 2011). In recent years, the association of biomimetic robots with living animals has seen a significant surge of interest, mainly in the fundamental scientific study of organisms. In addition to the basic research aspect, however, there could also be a far-reaching and even utopian aspect to this technology: Positively affecting a whole ecosystem’s stability by robot-organism-interactions, or, at least, slowing down the currently observed ecosystem decay this way (Lazic and Schmickl, 2021). This concept is inspired by a recent experiment that demonstrated an inter-species information flow mediated by robots (Bonnet et al., 2019). In this experiment, a fish swarm was connected to a group of honeybees, by integrating two types of biomimetic robots into these social animal systems and then connecting these robots *via* the Internet between Austria and Switzerland in a live group-coordinating data stream. In some sense, this experiment hinted how ecological linkages could be artificially created and showed that it is possible for two groups of organisms very distant from each other (spatially, developmentally, temporally, and on a size scale) to jointly make self-organized collective decisions, a process that can be interpreted as the first artificially created ecological interaction (Bonnet et al., 2019). We call this paradigm of ecosystem stabilization “Ecosystem Hacking” (EH) (Ilgün et al., 2021; Schmickl et al., 2021). The social integration of robots into animal societies is a form of “Organismic Augmentation” (OA), as it augments an existing animal superorganism with new capabilities, *e.g.*, new sources of information, that would be naturally inaccessible for these animals otherwise. Eusocial or social organisms are particularly suited to such an approach, as their social interaction mechanisms provide particularly rich behavioral interfaces for robots to “hook themselves” into the system, *e.g.* (Stefanec et al., 2021), and they are also usually of great ecological importance. Thus they are the most promising candidates for such an approach.

**FIGURE 1:**
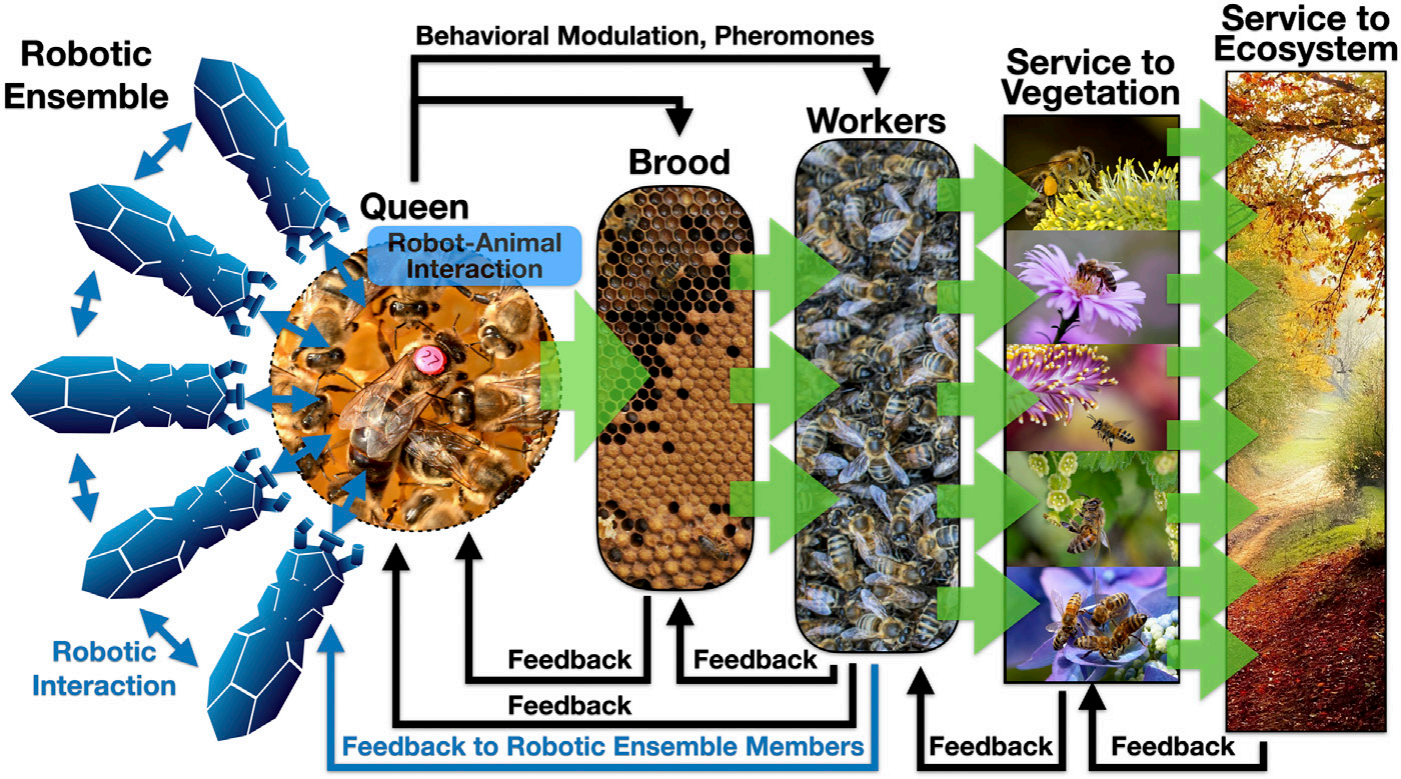
Basic concepts of a minimum-invasive form of “Ecosystem Hacking” and the associated/affected feedback loops. Blue thin arrows indicate interactions in which at least one partner is a robotic agent. Black arrows indicate the naturally present interactions. Green thick arrows indicate bio-mass production or facilitation of biomass production. Roundish shapes indicate regions inside of the colony, rectangular shapes indicate regions outside of the hive. All images used here are in the public domain.

The international EU-funded project RoboRoyale (2021) aims to explore such novel forms of ecosystem hacking, focusing its research on a minimally invasive way of EH through OA: By creating a set of robots that can specifically interact with one central organism in a honeybee colony: the honeybee queen. The goal of this technological approach is to influence the behavior of bees at the colony level, which ultimately affects the ecosystem in which the colony is embedded. This interaction with the queen will be achieved by a set of biomimetic robots, and will be purely based on social interaction behaviors (see Figure 1 for a schematic overview). Besides the depicted robot-to-queen interactions, also some robot-to-worker-bee interactions are planned to allow modulating pheromone transfer rates, however, our main focus will be on interactions with the queen.

Our study presented here is aimed to be one of the first showcases of the paradigm of OA. It suggests that social insect colonies are “augmented” by technological artifacts in order to become powerful biohybrid agents engaging in ecosystem stabilization. OA requires that the living organisms accept these technological units in their colony, so it is an invasive method for the superorganism as a whole, but it is not invasive at the organism level.

Technologies that integrate sensors into beehives are known as “Smart Hives” (Edwards-Murphy et al., 2016; Barlow and O’Neill, 2020), these technologies mainly focus on monitoring the bee colony. However, the OA paradigm goes significantly further so that it ultimately ends in developing an ecosystem-level effect: Specific biocompatible and biomimicking robots are integrated into the colony in order to modulate its collective behaviors. First steps have been made in this direction with robots imitating the waggle dance of honeybees (Landgraf et al., 2011). In contrast to one single robot, we work towards creating a full ensemble (team, group, swarm) of biomimetic robots that are accepted by the colony members, and especially by the honeybee queen. Successful integration of such a robotic system would also enable new possibilities for behavioral research within a superorganism: By controlling the robotic surrogates, the effects of different behavioral patterns on queen behavior and the colony itself can be explored. Ultimately, we hope to achieve a stabilizing effect on an ecosystem through this OA principle, which we categorize within the field of EH.

**FIGURE 2:**
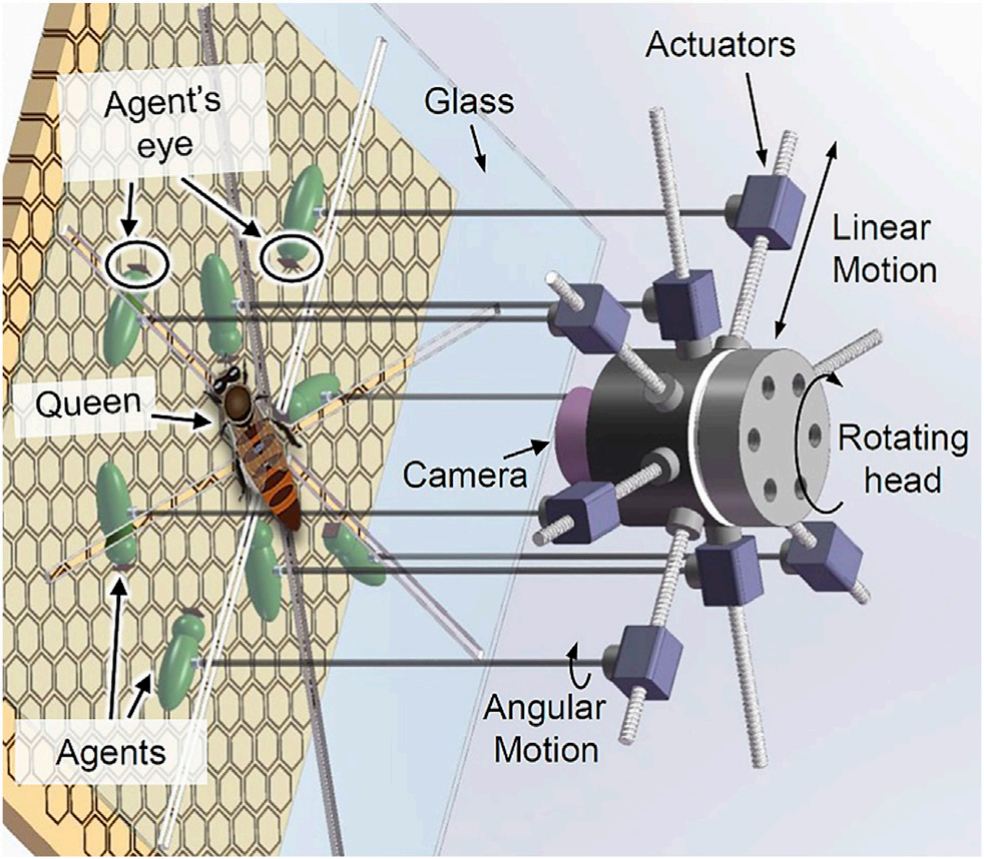
Internal structure of the robot with eight physical agents which can rotate and move forward/backward independently.

Figure 2 shows a basic concept for a physical robotic system, designed to interact with the queen bee. This robot is designed as a miniature manipulator that has eight arms. The end effector of each arm is a biomimetic agent, mimicking a worker bee acting as a queen’s attending bee. The micro-actuators of each arm can generate independent linear and rotational movement of the agents, while the entire manipulator can shift its position across the comb. A thin glass is placed between the comb and the main body of the robot so the queen’s and other bees’ movements are limited to a 2D space. The manipulator has a high resolution vision system that tracks the queen continuously and guides the entire structure in a way that the queen always stays at the centre of the manipulator’s operational space. The biomimetic agents will be equipped with microcameras, tactile sensors and actuators capable of cleaning, and providing food to the queen. Each agent will contain a microcontroller capable of handling basic data preprocessing and communication with a central control computer that is connected to the manipulator.

The main aim of the system is to interact with the queen *via* these biomimetic agents. Such an interaction will allow us to guide or to lure the queen to specific areas, affecting the distribution of the queen’s pheromones that impact the activity of the workers. Guiding the queen will also affect those hive regions where eggs are laid, and thus possibly optimize the distribution of the brood. In addition, the robotic agents will be able to offer a different food composition to the queen than the one that would be provided by the workers. *E.g.*, by providing the queen with more proteinaceous food, we can stimulate the egg laying activity, leading to more brood. This, in turn, will stimulate the workers to perform more flights, leading to stronger pollination of the surrounding ecosystem at the times determined by our system.

## 2 Materials and Methods

### 2.1 A Model of the Queen and her Court

Our approach aims to embed a set of biomimetic robots (see Figure 2) into the honeybee queen court system (see Figure 5). To be effective, these robots must not only be compatible with “court bees” in size, odor, taste, texture but also in their behavior towards their natural counterparts. As a first step, we want to understand the natural behavior of these court bees, to finally reproduce it on the robots. For this purpose, we developed a simple agent-based model of the movement behavior of the queen in the brood nest, as well as a simple model of the court bee’s movement behavior. This “queen and court bee” model, after being refined and validated by empirical data, will later be used to extract behavioral programs to be embodied by the ensemble of robotic agents that will physically interact with the biological queen and thus is a crucial first step towards a biohybrid agent for ecosystem stabilization. In the following we give a brief description of the model:

**Modeling the comb’s environment:** The brood area’s temperature field, shown as a green-to-white gradient in Figures 4A, is modeled by a simple diffusion process mimicking a temperature of 36–38 °C in its center and a temperature of lower temperatures (24–26 °C) on the outer rim. Agents move in continuous space and the static temperature field is modeled in discrete patches of 1 × 1 cm (*T*_*X*,*Y*_). Besides the static temperature field, the model also depicts a dynamic pheromone field, as the queen is emitting pheromones (QMP, see Hoover et al., 2003) that spread with the contacts of the bees (not modeled here yet) and by basic physical processes (modeled here). We model a steady emission rate of *E*_*pheromone*_ = 10 pheromone units per time step, a steady proportional decay by *μ*_*pheromone*_ = 5*%* per time step and a standard diffusion process with maximum diffusion coefficient *D*_*pheromone*_ = 1.0 per time step. This dynamically changing pheromone field is modeled as a set of variables of *P*_*X*,*Y*_(*t*) for every patch in the model. See Figure 4A for a depiction of this field around the queen (purple shades).

The model tracks the behaviors and states of a population of honeybees in a colony *C* = {*Q*, *W*}, where *Q* is a queen agent and *W* is a set of worker bee agents *W* = {*W*_1_, *W*_2_, … , *W*_*N*_} of size *N* = 100. All agents perform a sequence of behavioral actions per time step, as described below.

**Orientational behaviors:** In order to model a positive thermotactic behavior that keeps the agents within the warm brood area, all agents make a turn towards the warmest neighboring patch with a probability *p*_*thermotaxis*_ = 0.125 per time step if the local temperature *T*_*X*,*Y*_(*t*) of agent *i* at time *t* is below a threshold of *Θ*_*temp*_ = 1.0, which is representing the brood area’s outer rim temperature at approx. 30–32 °C in our model. In addition to that, a worker bee agent *i* that is currently responding positively to the queen’s pheromones (*P*_*X*,*Y*_(*t*) ≥Θ_*taxis*_ (*i*, *t*)) will turn towards the neighboring patch with the highest value of the pheromone field (positive chemotactic orientation). In contrast to that, if an agent *j* experiences a pheromone concentration below its behavioral threshold (*P*_*X*,*Y*_(*t*) < Θ_*taxis*_ (*j*, *t*)), it will turn to the patch with the minimum pheromone concentration (negative chemotactic orientation), but only if the pheromone is above or equal to a minimum sensory threshold (Θ_min_ = 2.5 pheromone units), otherwise these agents will turn only randomly.

**Random turns:** After the execution of orientation behavior, all agents perform a correlated random walk with a probability *p*_*Turn*_ = 0.6 per time step. A random rotation occurs that deviates their direction by a uniform random value within ±15 degrees of their current heading, thus modeling the orientation behavior with a slight random movement.

**Motion behaviors:** With a probability of *p*_*move*_ = 0.3 per time step, agents will move forward in their current orientation. The queen agent moves forward with a speed of *v*_*Q*_ = 0.9 cm per time step, while worker agents in the agent set *W* move forward with a speed of *v*_*W*_ = 0.6 cm per time step. Agents can only move if their path (in front of them) is not already occupied by another agent, thus physical blocking and the resulting crowding effects are modeled.

**Model of reversible habituation mechanisms:** Natural court bees are known to show a specific behavior of switching between positive and negative attraction to the queen: They first stay with the queen for a significant time, in order to lick, clean and feed her, while taking up many chemical compounds from the pheromone mixture that the queen displays and emits. Then, they move away from the queen for a significant time period during which they run all around the hive. It is assumed that this way the chemicals that indicate the presence of the queen are distributed across the hive (Seeley, 1979). In order to model this behavior we employed the “threshold-reinforcement mechanism” (Gautrais et al., 2002) in our model. This mechanism is known to be capable of yielding the spontaneous emergence of specialized behaviors (e.g., queen court attendance here). We extended this mechanism such that it is capable of reproducing this behavioral specialization in a rhythmic on-off pattern.

**FIGURE 3:**
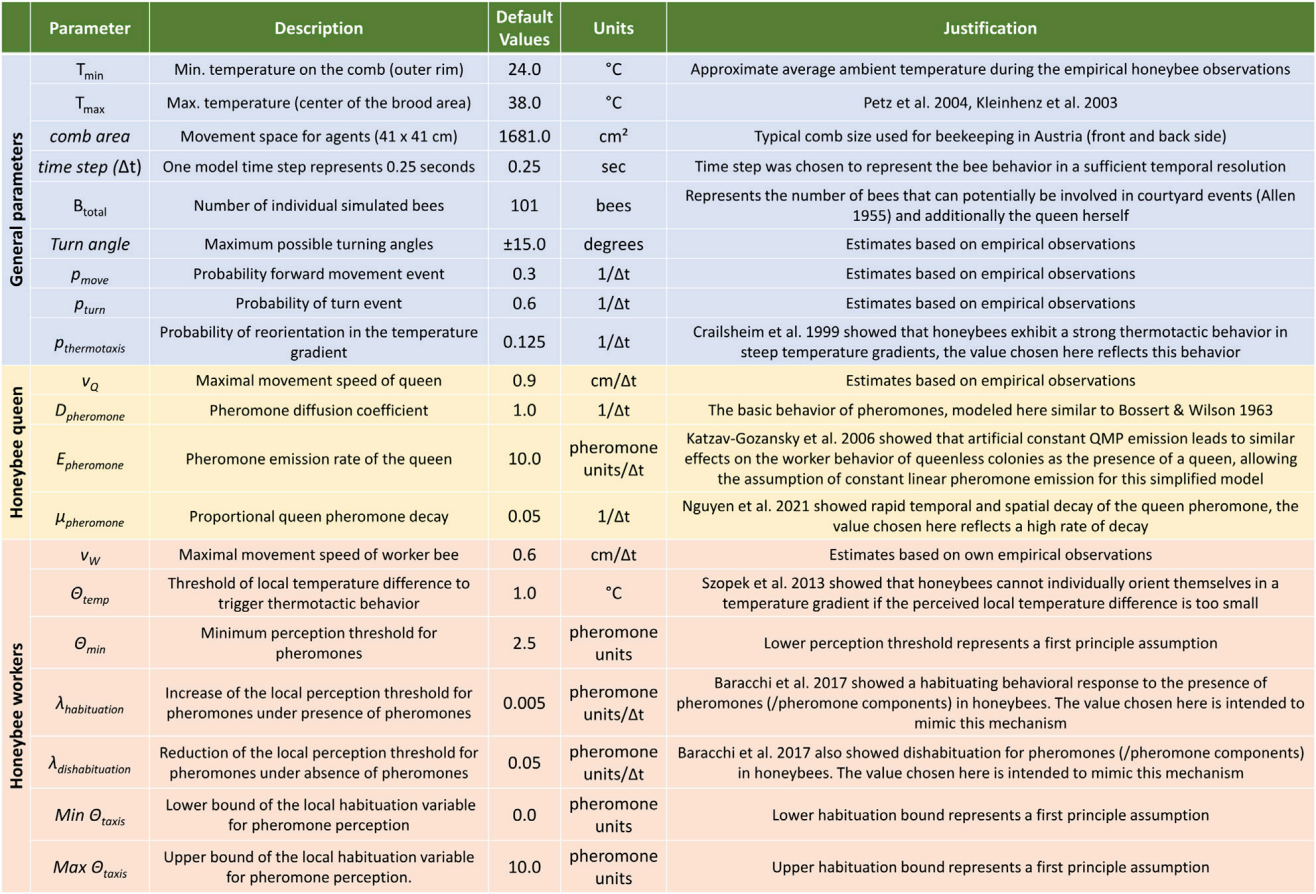
Summary table of model parameters. Parameters could be derived (either quantitatively or qualitatively) from other research publications (Allen. 1955; Bossert and Wilson, 1963; Crailsheim et al., 1999; Kleinhenz et al., 2003; Petz et al., 2004; Katzav-Gozansky et al., 2006; Szopek et al., 2013; Baracchi et al., 2017; Nguyen et al., 2021), collected *via* empirical observations, or originate from first principle assumptions.

Θ_*taxis*_ (*i*, *t*) governs the worker’s behavioral response to pheromone presence, if it is above the sensing threshold. At every time step, a worker *W*_*i*_ reacts to the pheromone with positive chemotaxis (uphill walk), the threshold Θ_*taxis*_ (*i*, *t*) is increased by a value of *λ*_*habituation*_ = 0.005 pheromone units per time step. This makes it harder to trigger the behavior again, but the positive chemotaxis increases an agent’s probability of residing in a region with higher pheromone concentrations. Alternatively, if no pheromone was sensed above the sensing threshold, Θ_*taxis*_ (*i*, *t*) is decreased by a value of *λ*_*dishabituation*_ = 0.05 per time step. The adapted values of Θ_*taxis*_ (*i*, *t*) are bound to the range of 0.0–10.0 pheromone units. All model parameters have been summarized in Figure 3 and further explanations and references for the choice of each parameter have been included.

**FIGURE 4:**
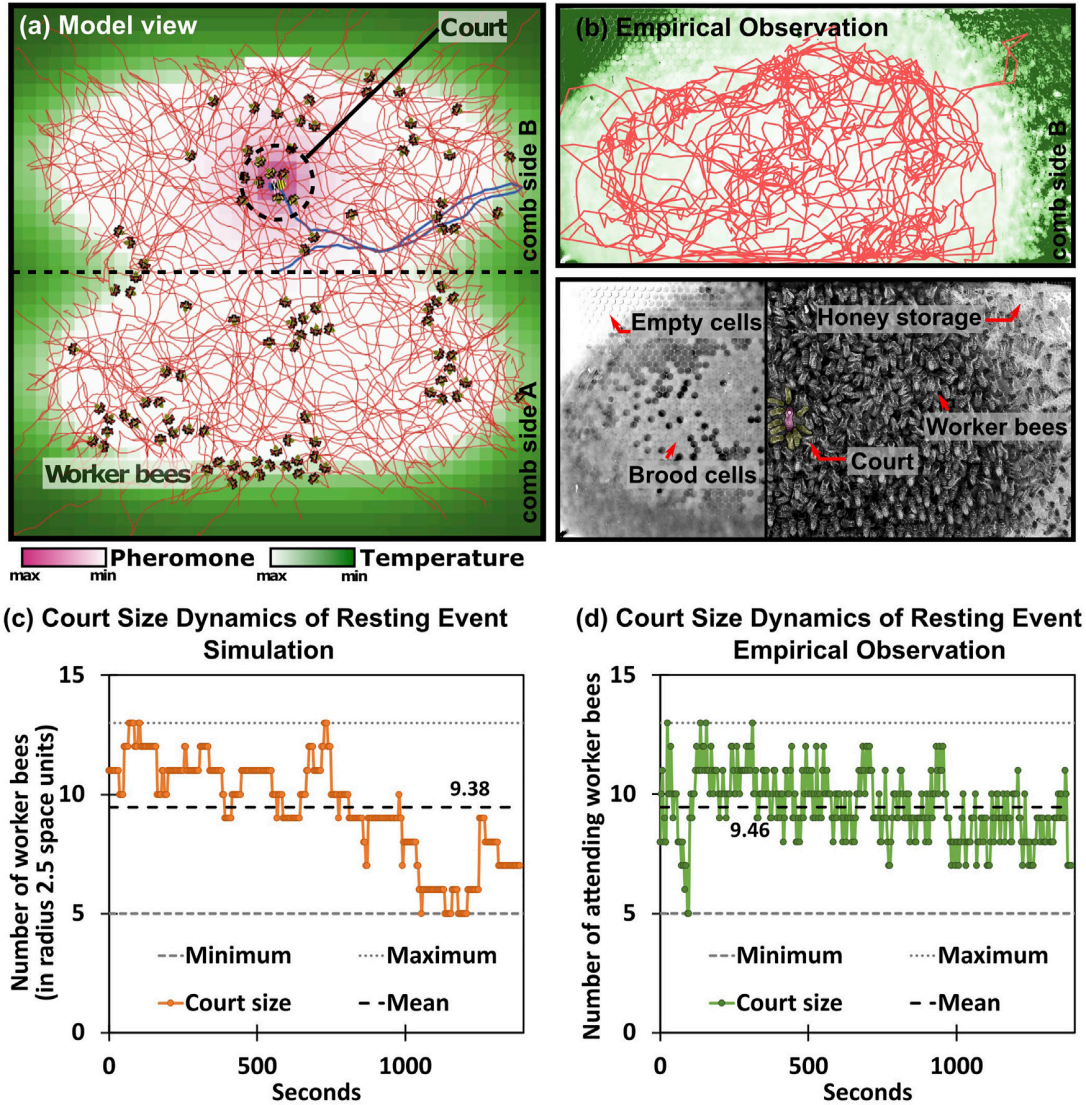
General model overview. **(A)** Spatial representation of the queen-court-broodnest system in our model. Shades of green indicate the underlying static temperature gradient in and around the brood nest area of the comb. We modeled two comb sides, the place of potential crossing between comb sides is indicated by the central horizontal line. Shades of purple show the pheromone gradient emitted by the queen, lines indicate agent movement patterns. **(B)** Upper sub-figure: Empirical observation of queen movement pattern (red line) over the course of 6 h in an observation hive, shades of green indicate the area of the brood nest that normally corresponds to a temperature gradient. Lower sub-figure, left: Underlying honeycomb structure made visible by computer-assisted removal of moving bees in the foreground, light cells in the center of the honeycomb represent capped brood cells; right: image taken from an observation hive with highlighted queen and queen-court **(C,D)** Emerging queen-court size dynamics during an exemplary queen resting event in the model **(C)** and empirically observed **(D)** over the course of 1,400 s, court size varies between 5 and 13 worker bees with an arithmetic average of 9.38 bees (model), and 9.46 bees (observation).

This implementation of the individual adaptation can be interpreted as an inverse version of the known “threshold-reinforcement mechanism” model (TRM), which is known to yield individual task specialization. It is an “inverse” of our model here, because the two threshold adaptations are with opposite signs compared to our model here, thus the TRM model is imposing a strong self-reinforcing positive feedback loop. In our implementation, the inversely signed *λ* coefficients of adaptation lead to more complex behavior: Agents can initially specialize into “court bees” by actively searching for maxima in this gradient. Then they will habituate over time and consequently move away from the queen until they have become receptive again for the chemical queen stimulus due to dishabituation. Such behavior emerges from our model in the form of a fairly homogeneous spatial coverage of the non-court worker bees over the entire brood nest area (indicated by the thin red trajectory lines in Figure 4A), as well as the Figure 4C dynamics of the court site (as shown in Figure 4C in comparison to an exemplary observed queen court event Figure 4D).

### 2.2 Model Validation

From recordings of honeybee observation hives, we selected three times 60 h (from August 2019, 2020 and 2021) from three different hives and manually tracked the queen’s movement using a “click-script” (written in Python/OpenCV). The exact setup and the camera model^1^ varied slightly between the years, but all were recorded under IR light (which can not be perceived by bees) and photos were taken every 4 sec (see exemplary image in Figure 4B). On the surface of the combs, 1 mm corresponds to ≈5 pixels (px).

To filter the data for potential “queen court events” (QCE)—where the queen is at rest and surrounded by the court worker bees—we defined two thresholds: We record a QCE, when the queen’s walking speed is less than 1 px/sec (≈0.2 mm/sec) for longer than 48 sec. A total of 443 court events were detected that meet these criteria. For each such event, all corresponding photos including the unmarked frames in between were cropped to 360 × 360 px around the queen’s average position. For manual counting of the attending court bees in all events, we selected a cropped photo halfway into each event.

## 3 Results

**FIGURE 5:**
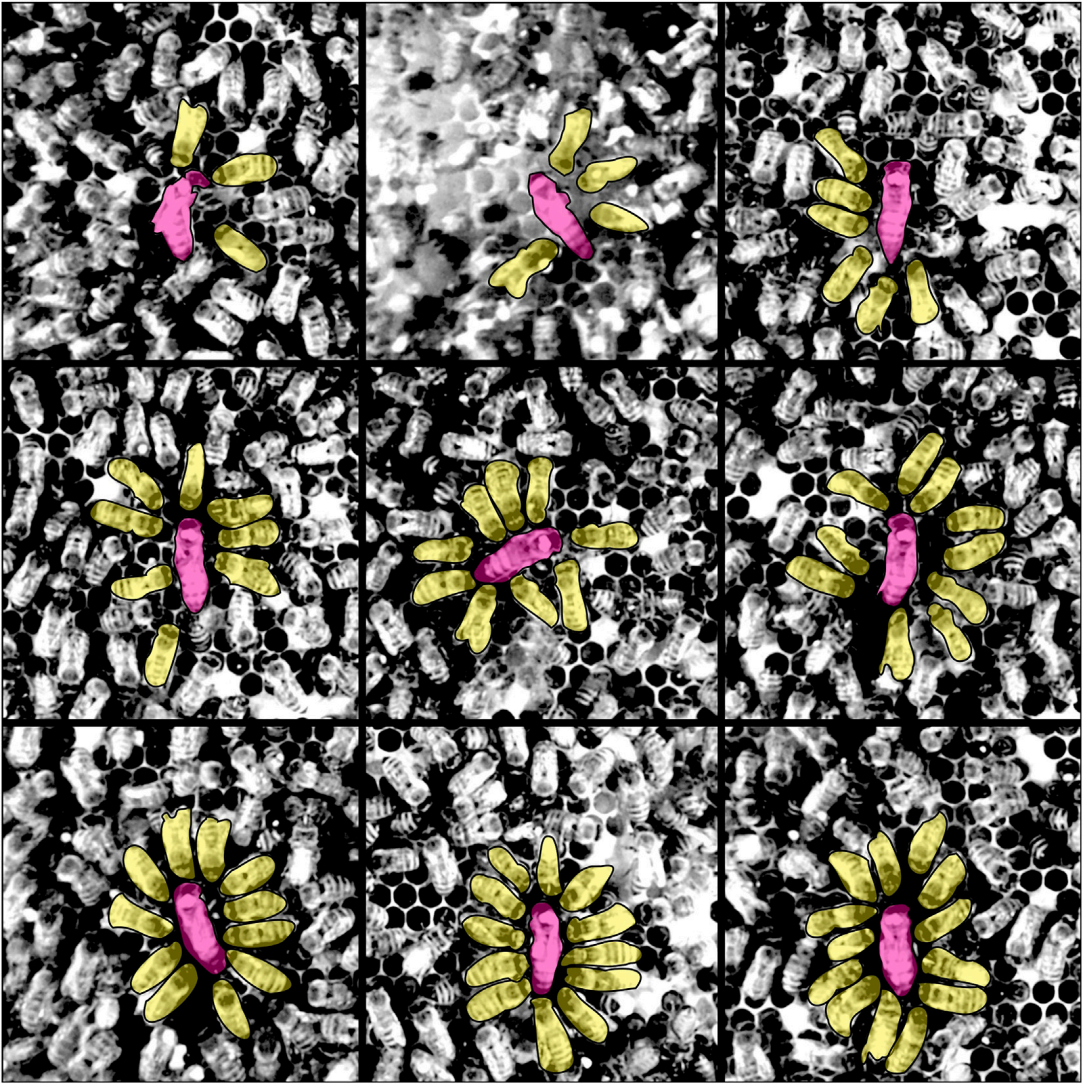
Sample observed queen court events of various sizes. 9 exemplary queen court events with increasing number of attending bees from top left to bottom right. The queen (pink) and the court worker bees (yellow) are highlighted to indicate the court visually.

**FIGURE 6:**
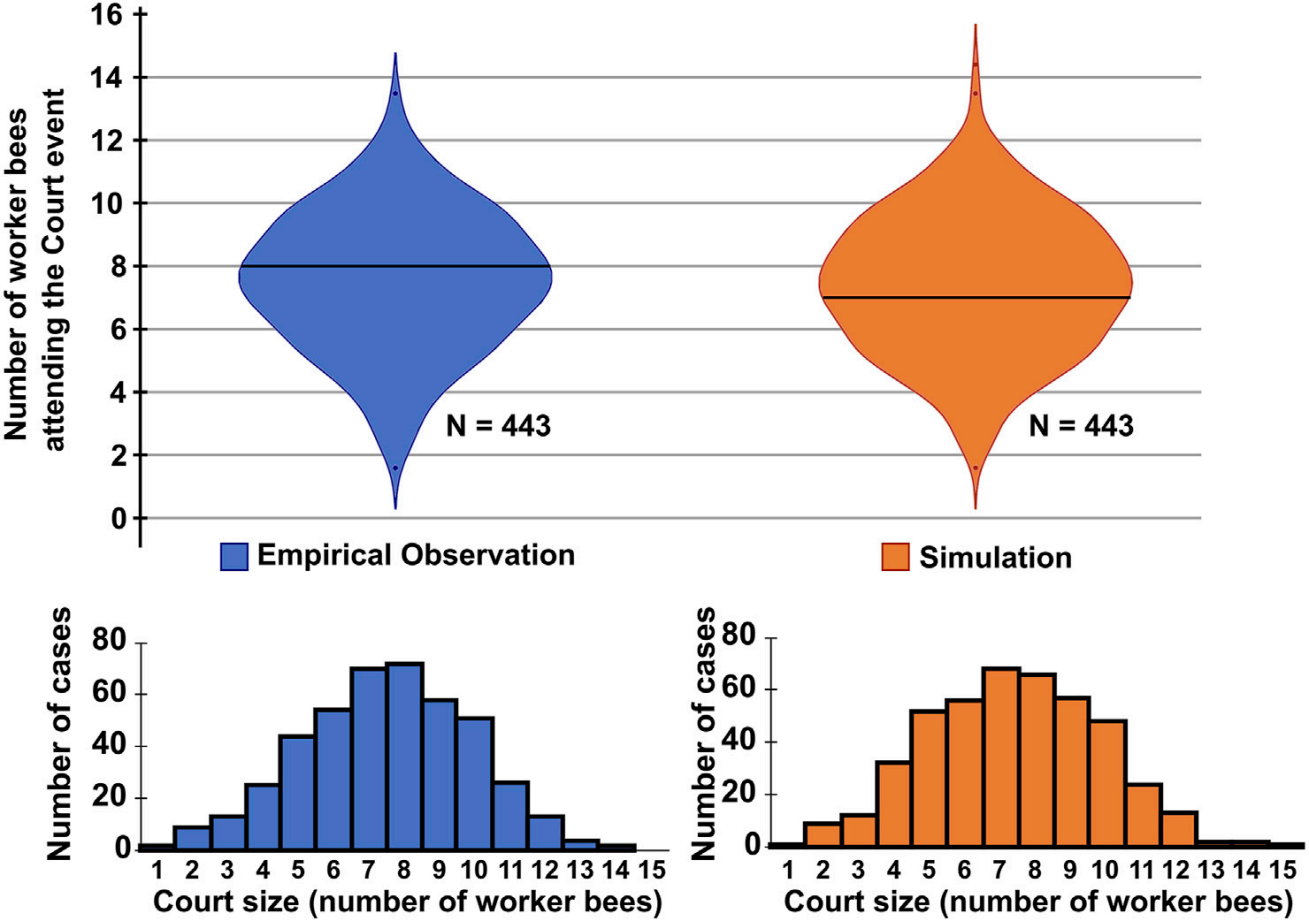
Violin plots and histograms for comparing emerging court sizes of empirical observation (left, blue) and model simulation (right, orange). For empirical data, 443 queen resting events of 180 h of video recording were manually detected and the size of the court was determined at the middle of the event. For the model simulation, 443 queen resting events were simulated and the number of worker bees within a radius of 2.5 space units around the queen was counted at the middle of the event. A two-tailed Mann-Whitney *U* test for difference between the two data sets revealed no significant difference (*p* ≈ 0.35).

The implementation of the model is simple, but, as shown in Figures 4, 6, it already provides qualitative and quantitative results. We gathered empirical data and manually detected 443 court events over the course of 180 h of video footage from three different observation bins spanning 3 years. Individual events last for 48 sec to 30 min, with the majority of event frames showing the queen surrounded by her court. Figure 5 shows a sample of 9 queen court events with increasing number of attending bees. For easier visibility, the queen and the workers involved are highlighted. The distribution of sizes of the court in these events are shown in Figure 6.

The queen court emerges at various places of the brood nest area of the comb, where it forms as an assembly of a set of worker bees that place themselves around the queen, orient towards her and stay in this position for longer times. Also in our model such dynamic courts of comparable sources emerge, see Figure 4.

In the empirical study by (Seeley, 1979), it was reported that the queen performs egg-laying steps over an extended period of time, during which she does not move much in space, alternating with periods when she moves longer distances across the brood nest area. During the periods when the queen tends to stay in one place and may only move a little locally, we also see that larger and more stable court emerge during these periods, which is also reported in the literature. We use these phases here to quantitatively validate our model, as these phases can be detected in manual review of video footage due to the prolonged immobility of the queen and the court amidst the near chaotic movements of the surrounding bees.

We measured the sizes of the queen’s court during this period of time and found that the courts that emerge in our model compare very well to court sizes observed empirically, as is shown in Figure 6. For the resting periods of the queen, our model predicts emerging court sizes of, on average 7.4 bees (Median: 7 bees, IQR: 3 bees). In comparison, in 443 observed queens resting phases, on average a court size of 7.5 bees was observed (Median: 7 bees, IQR: 3 bees). A two-tailed Mann-Whitney *U* test comparing the two data sets failed to detect a significant difference (*p* > 0.35).

In addition to the resting phases of the queen, there are also repeated movement phases about which our model can make predictions: Over the course of about 9 days of simulation, the queen spent only approx. 7.9% of her time budget in displacement behavior (walking longer distances, more than 5 consecutive translocations in a row) and the rest of the time the queen either fully stopped for longer times (resting) or is wiggling around within a small area (egg laying like motion behavior). These two behavioral patterns are not yet distinguished in our model because the model does not include egg-laying and emerging brood patterns so far. We summarize both behaviors of non-significant dislocation as one state named “resting” here. This simulation dataset is in close resemblance to the reported empirical data (Seeley, 1979), which reports the queen’s time budget of longer-distance traveling to 5.4% of the observed time, which is not far away from the 7.9% that emerges in our simulation. Given the simplicity of our multi-agent model, we interpret this as a good model validation.

**FIGURE 7:**
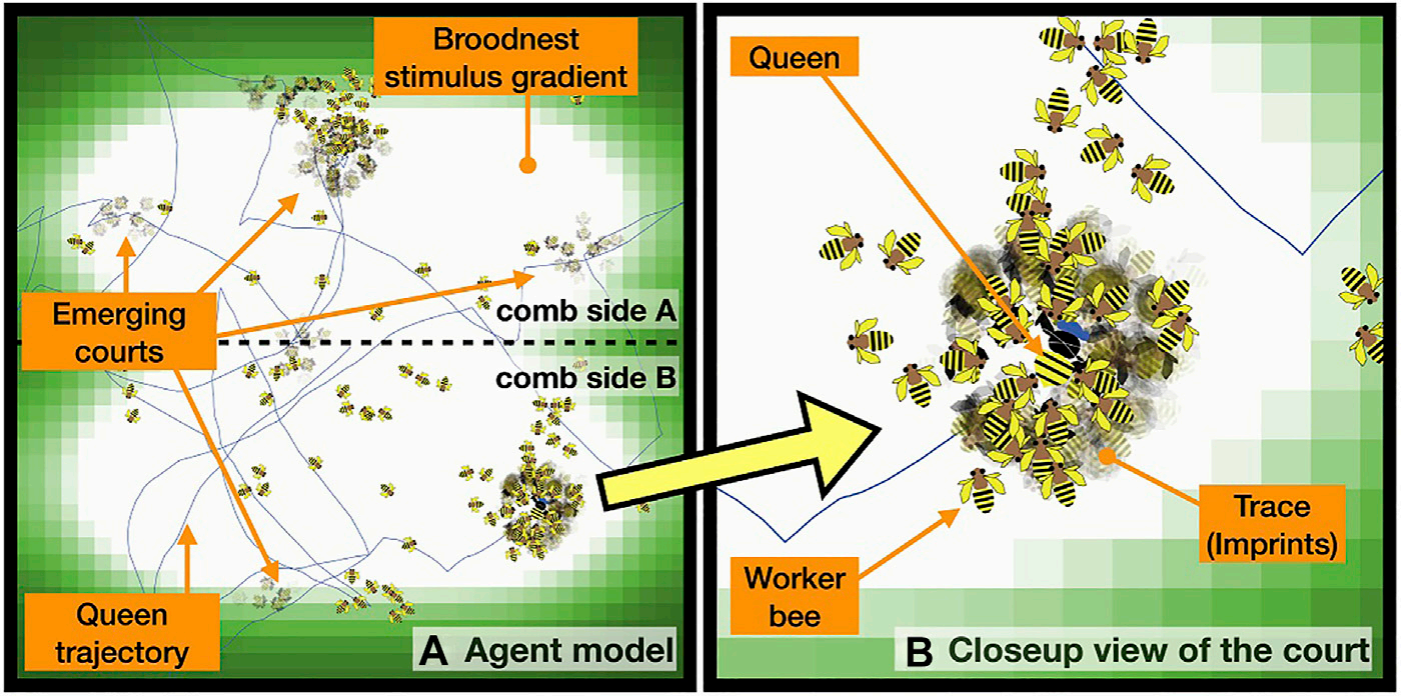
**(A)** Queen trajectory in a typical model run, imprints of body shapes indicate places where worker bee agents stood still for longer periods of time in the vicinity of the queen, indicating past court events at these places. **(B)** Close-up view of the court region.

Figures 7A,B show a qualitative similarity to what is observed from the natural court bee behavior, *e.g.*, with regards to the dynamic courts as described in the literature from the observation of natural honeybee court systems (*e.g.*, Seeley, 1979; Free et al., 1992; Schmickl et al., 2003) and our own observations reported in Figure 4.

In order to study the system in more depth, we conducted a series of simulation experiments to further validate the model against the empirical behavioral data on the queen court.

Concerning spatio-temporal features of the court, a similar picture also emerges in our simulation, as is shown in Figure 7, where we visualize the dynamics of the worker bees around the queen by making them “stamp” their body-shapes in a semi-transparent way onto the simulation’s background image, which indicates also the temperature gradient of the brood nest area. All worker agents located within a radius of *r* = 2.5 cm around the queen, “stamp” (copy) their body shapes onto the background map with a very low alpha-value (almost transparent) every 5 sec. In consequence, stronger imprints appear where the workers have performed this stamping often at the same place, thus this indicates that they resided there for longer times. If this imprint is dark but shows no sharp shapes, an agent either has either wiggled in place, or there were even many agents that appeared in a coming-and-going pattern. If an imprint is strong and clear, it indicates that one agent stood in place without motion for a longer period of time (Figure 7).

**FIGURE 8:**
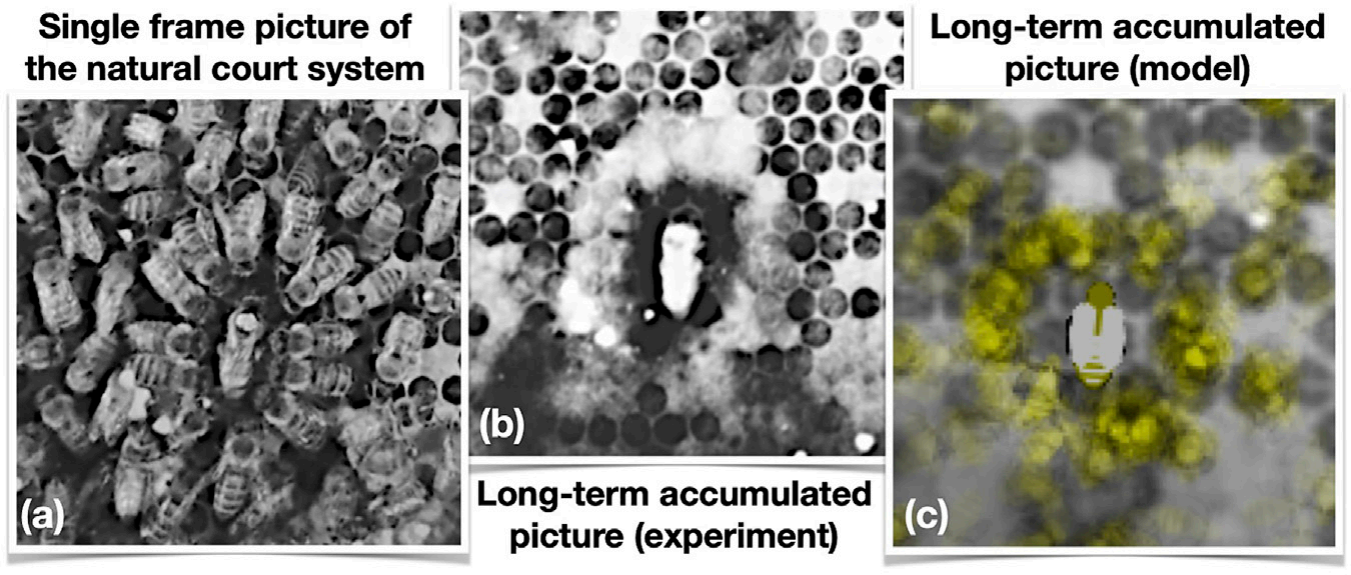
A queen with her courtyard. **(A)** Photo shot in IR light, single frame from a courtyard event. **(B)** Median of 154 photos across the duration of the same event, histogram-equalized. **(C)** Accumulated picture of a simulation run of our model in a similar courtyard event. Original extracted background of comb was projected into the simulation background, worker bees left a semi-transparent yellowish body imprint every 30 s, forming a halo-like structure around the queen, similar to empirical findings shown in **(B)**.

In order to further compare the court patterns that emerge in our agent simulation to naturally observed court events, we conducted an additional analysis on the cropped photos of the 443 detected court events (see section “Observing the Queen Court”). The stack of all grayscale images of an event is normalized to the global minimum and maximum pixel intensities and the median intensity is computed for every pixel across the event. This procedure highlights the queen and her court, while randomly moving bees mostly vanish. Lastly, the resulting image is histogram-equalized (see Figure 8B).

In order to qualitatively compare that with a simulation run (Figure 8C), we ran our model in a simulation setting with 100 bees and 1 queen. To emulate a similar situation as is shown in Figures 8A,B, we imported an extracted background image from the videography processed from that very same day. Then, we placed the queen at the initialization of the simulation at a location corresponding to the courtyard event shown in Figures 8A,B with similar orientation onto that background image. The runtime of the simulation was 100,000 time steps (approx. 7 h). Every 30 simulated seconds, the simulator checks whether or not a worker bee has been staying in place for 30 sec (or more), rotations at the same place were allowed. If a worker bee did not walk during this interval, it stamps its very pale yellowish body shape with a very low alpha channel value, thus in a semi-transparent way (RGB = {1, 1, 0}, *α* = 7).

Comparing Figure 8C to the empirical data shown in Figures 8A,B, a striking resemblance can be found: Around the queen, several worker bees stay in place for longer times while they are part of the court and thus they leave behind a halo-like structure on the background image slowly over time. In contrast to that, at places further away from the queen this happens more rarely, creating just some very thin fog-like traces on the background image.

### 3.1 Model Results

**FIGURE 9:**
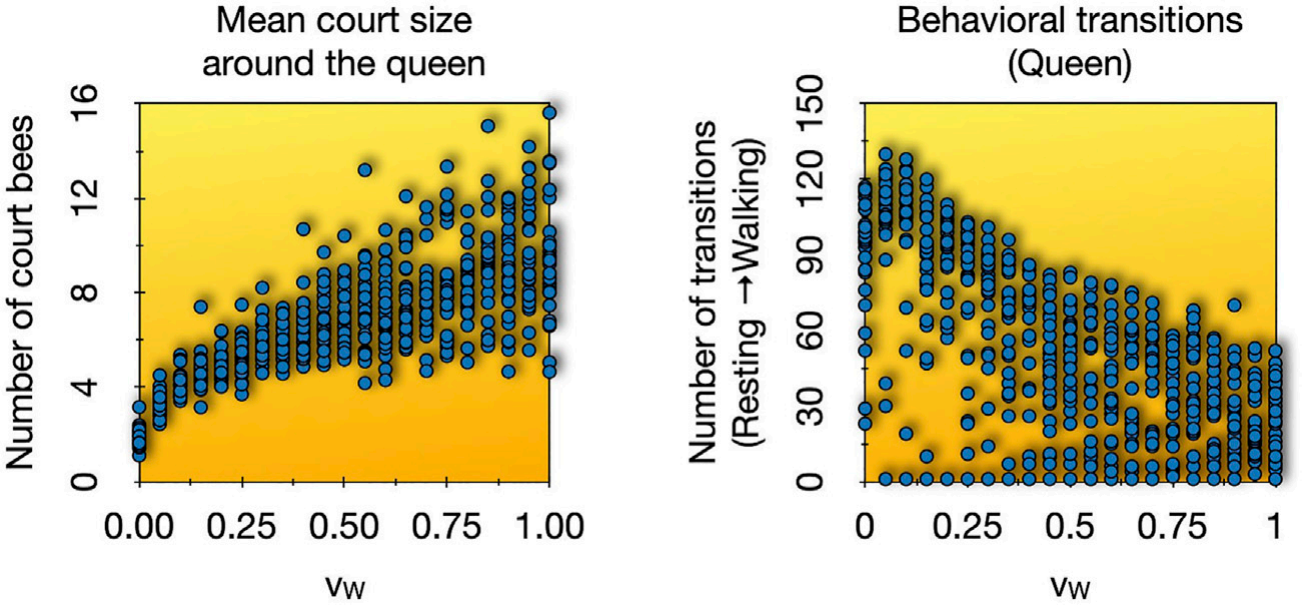
Parameter sweep over worker bee speed (*v*_*W*_) from 0.0 to 1.0 in 0.05 intervals, 30 repetitions each. Shown are the final values after 125 min in the observed variables. Left: The mean values of court sizes per run. Right: The number of transitions from resting state to walking state over the runtime of each experiment.

In order to examine our model’s capabilities, we performed a parameter sweep of the parameter *v*_*W*_ to see what effect this microscopic motion-related parameter has on the phenomena that arise on the macroscopic system layer. We conducted 30 repetitions for each value of *v*_*W*_ between 0.0 and 1.0 in steps of 0.05 intervals. We found a positive sublinear correlation between the predicted size of the queen court, that emerges in the system, with increasing worker bee speed. This in turn seems to affect the behavior of the queen herself in a negatively correlated way: The faster the worker bees are, the more massively the court can grow, causing a decrease in the predicted frequency of the queen’s switches between walking and resting phases (Figure 9). The queen was considered to be resting if she did not walk forward for 5 or more time steps. Resting ended as soon as she moved forwards in one time step. Our results show that simple microscopic parameters, like the worker bee speed, may not only affect macroscopic phenomena but may also elicit significant behavioral modulations. Court size, worker turnover and the queen’s motion across the hive are highly relevant factors for transmitting and depositing pheromones in the colony, a crucial factor in a colony’s self-regulation. Our model suggests that subtle changes or modulations in the individual behavior of court bees (microscopic system level) can already have significant macroscopic effects on the colony level, while at the same time Figure 9 shows that the model is not overly sensitive in the way how macroscopic traits depend on microscopic mechanistic parameters.

## 4 Discussion and Conclusion

### 4.1 Ecosystem Hacking

**FIGURE 10:**
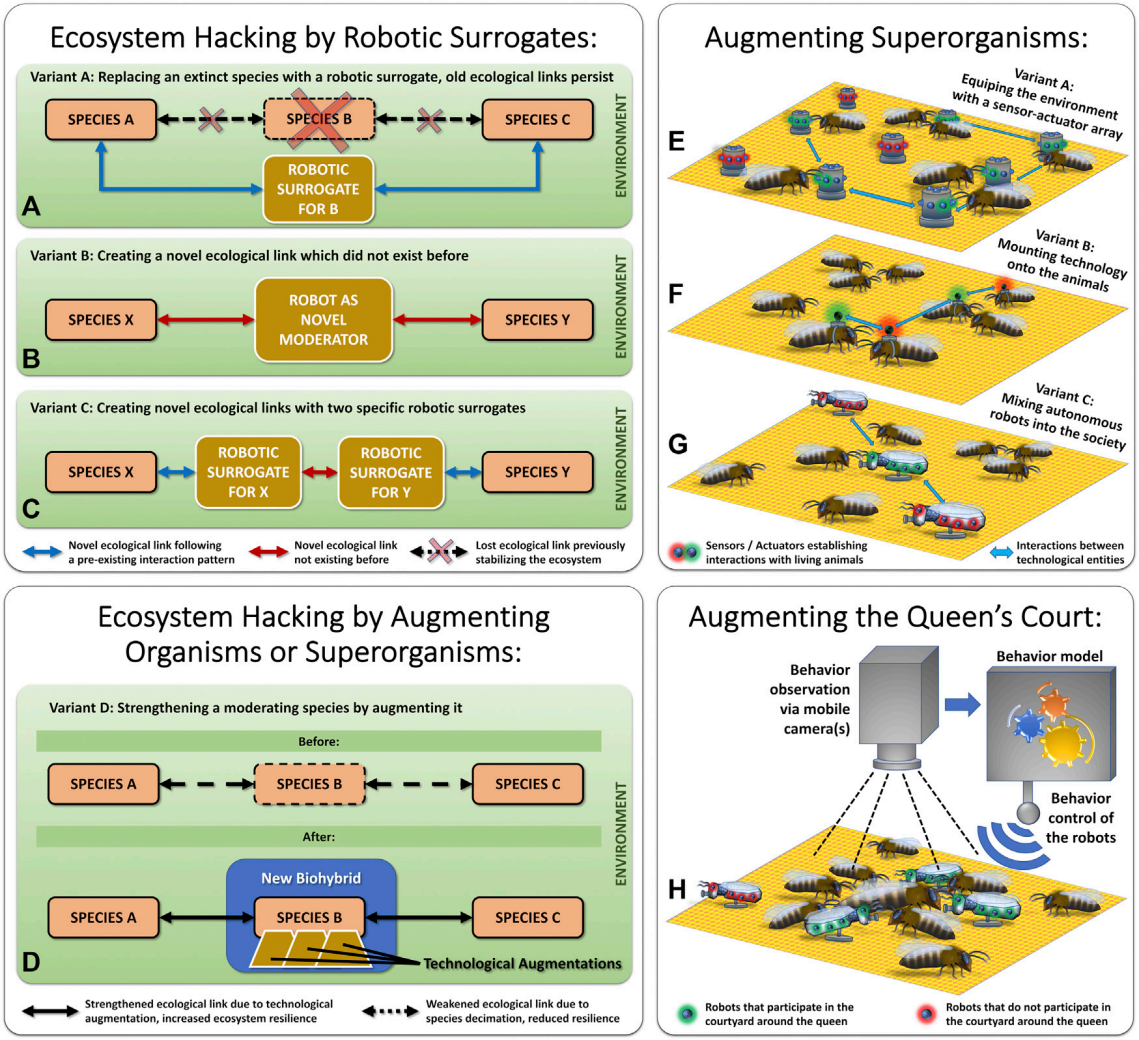
Variants of “Ecosystem Hacking” and “Organismic Augmentation”. Left top subfigures **(A–C)** show variants of ecosystem hacking approaches that rewire ecosystems. Right top subfigures **(E–G)** show variants of organismic augmentation for superorganisms or other social animals. The left lower subfigure **(D)** depicts how existing ecological conditions can be strengthened by augmenting a central keystone species. The right subfigure **(H)** depicts augmenting only a central subelement of a superorganism, the queen’s court.

The term “Ecosystem Hacking” refers to a concept aimed at stabilizing endangered ecosystems by introducing technology (e.g., sensor-actuator nodes, robots) as actively participating components within these ecosystems. There are several variants for such an endeavor presented in the literature, the study at hand adds the specific method of augmenting a honeybee queen’s court as a novel method to this collection. Figure 10 gives a graphical overview of the diverse variants for ecosystem hacking, depicting already discussed variants from literature in its upper subgraphs and depicting the novel variant presented in the study at hand in its lower subgraphs.

In a recent review article (Szopek et al., 2021b), three principle methods for ecosystem hacking are discussed, these are visualized here in Figures 10A–C: 1) Replacing an organismic species, that has already gone extinct, by a tailored biomimetic robotic surrogate. In this case, the original species serves as a blueprint for designing the robotic surrogate. 2) Re-stabilizing an ecosystem, after significant destabilization has happened due to diversity losses, by establishing novel ecological links, that did not exist before. This method “rewires” the ecosystem in order to stabilize it. In this case, there exists no blueprint for the robotic agent, it needs to be designed from scratch with a decent ecological understanding of the specific ecosystem. 3) As a combination of the two before-mentioned approaches, a novel linkage in the ecosystem can also be created by first mimicking two natural species in a biomimetic way with two specific robotic surrogates and then creating a novel interaction between these two surrogates.

Here, we suggest performing an alternative and more proactive approach to ecosystem stabilization. Instead of acting after a diversity loss has already happened, we suggest supporting important keystone species (e.g., social insects) by proactively strengthening them with modern technology. A promising approach is to first select an endangered keystone species and then use technology to support it and prevent its decline, as it is depicted in Figure 10D. Very often, eusocial insect colonies are such keystone species, and their mechanisms of social integration offer a plethora of “social hooks” to allow technology to integrate itself into the collective. An insect colony is called a “superorganism” because the whole colony acts and replicates like it was one single organism, where the individual animals (workers, drones, queen) cooperate like the individual cells in a human body.

The approach to incorporate supportive technology into social insect colonies, or into other animal societies, is called “Organismic Augmentation” (Schmickl et al., 2021; Ilgün et al., 2021). The research project RoboRoyale works towards augmenting superorganisms with supportive technology in a novel way, by concentrating on the honeybee queen. This is different from the methods employed in the research project Hiveopolis (Ilgün et al., 2021) and ASSISIbf (Schmickl et al., 2013a,b) where worker bees are the target animals. Another, yet very different, take on organismic augmentation is made by the project Robocoenosis (Rajewicz et al., 2021) by starting with the technology first and then integrating living organisms into this technology, this way replacing robotic components like sensors or actuators step by step. All these approaches differ significantly in their methods; however, they have a very similar outcome: They create a novel biohybrid system that interacts with other components in its environment, this way the biohybrid system becomes an active agent in its ecosystem.

How the technological augmentation of superorganisms, for example, honeybee colonies, can be achieved to create novel biohybrid entities has been demonstrated in various research projects in the past (Schmickl et al., 2021). Figures 10E–G depict which methods are applied most often:

1) The technological components can be added to the environment, forming a sensor-actuator-array, in which static technological nodes can sense and affect the animals, while the technological nodes can also communicate with each other and, for example, self-organize spatially this way. This was demonstrated in the research projects ASSISIbf (Schmickl et al., 2013a,b) with a so-called “CASU” array (Griparić et al., 2017) and in the research project Hiveopolis with an augmented honeycomb (Ilgün et al., 2021; Stefanec et al., 2021) as well as in the research project Flora Robotica (Hofstadler et al., 2017; Wahby et al., 2018) with a group of growing plants.

2) The technological elements can be autonomous mobile robots that integrate themselves into social groups by interacting with living organisms. This was demonstrated in various studies with honeybees (Lazic and Schmickl, 2021; Landgraf et al., 2010, 2011), in several fish-and-robot setups (Faria et al., 2010; Landgraf et al., 2016; Bonnet et al., 2017; Bierbach et al., 2018; Bonnet et al., 2018) and in the research project Leurre with cockroaches (Halloy et al., 2007).

3) Finally, it is also possible to mount technological devices onto the animals, and even to affect their behaviors this way. This method was demonstrated for sensing purposes with honeybees carrying RFID chips (He et al., 2016), and for actuation with technologically augmented cockroaches (Sanchez et al., 2015). Closed-loop control between sensing and actuation was demonstrated with a herd of cows in which the cows carried augmentation devices around the head (Correll et al., 2008).

The European honeybees (*Apis mellifera*), as a major keystone species and very important pollinators (Hung et al., 2018), have been in a drastic decline for more than a decade (Hristov et al., 2020). In the study at hand, we investigate the first steps towards a novel approach to integrate technology into this type of superorganism, as it is shown in Figure 10H: By augmenting only one central and small group of animals, the queen court, we aim at a minimally invasive method of organismic augmentation. A few mobile robotic agents integrate themselves into the queen court and support this very central structure in the otherwise quite decentralized self-organization and self-regulation of the colony. These agents are driven by algorithms that were extracted from behavioral observations and behavioral models of natural courtyard bees; thus, they are biomimetic agents also concerning their behavior. First, they will integrate seamlessly, then they can, *via* subtle modulation of their behavioral parameters, also exert some influence onto the queen court, which in turn will influence the queen, and ultimately, will influence the whole colony in the long term.

There are many beneficial services that such a technologically augmented queen court can provide: Modern technology (e.g., robots) have access to information unavailable to the bees themselves, for example, weather forecasts can be acquired from the internet. This information can be utilized by the robotic agents in two ways: On the one hand, up to 95% of the brood, that is hatching from the queen’s egg-laying, is lost in times of bad weather periods. Thus, timely down-regulating the queen’s egg-laying activity will be beneficial for a colony’s efficiency, as brood that is not expected to survive will not be produced, to begin with. This way, important energy and nutrient reserves of the colony can be saved. On the other hand, the weather also affects the flowering, blossoming, and nectar-secreting activities of plants. Thus, timely up-regulating the brood production will also increase a colony’s efficiency in foraging and, ultimately, increase the ecosystem service that can be provided through pollination flights. Besides the brood and foraging, there are other important aspects where the robotic agents can play a beneficial role: In the reproductive cycle of honeybees, swarming is a critical and risky event. Thus, monitoring and regulating the “swarming mood” of the colony, by observing (and potentially modulating) the queen’s behavior, accordingly, may prevent colonies from splitting in unfavorable times. In addition, health observations of the queen and applying specific food, or even medicine, through the natural channel of mouth-to-mouth feedings, can further enhance the survival of augmented colonies. Figure 1 shows how modulating one single organism, the queen, with only a small ensemble of autonomous robots, can unfold its effects in a cascading way: first, the brood production is modulated, then the workers that hatch from the brood are affected, then the plants that are specifically visited by forager bees’ profit and finally the whole ecosystem profits, as many species interact with these plants directly or indirectly.

### 4.2 The Court Model

This study also reports empirical observations we made about the queen court dynamics in a honeybee colony. The court size we determined corresponds to similar results from previously published studies (Velthuis, 1972; Free et al., 1992; Schmickl et al., 2003). Based on our own empiric observations and on the data that is reported in the literature (e.g., Seeley, 1979; Free et al., 1992; Schmickl et al., 2003), we created a simple multi-agent model of the queen and her court bees.

The design goal for the model presented here is to be able to capture the observed emerging collective behaviors of the courtyard bees and the emergent path trajectories of the queen, which arise from queen-to-worker-interaction, by a model that is as simple as possible. This not only follows a good rule of model building, known as the “parsimony principle” (Gauch Jr et al., 2003), but such a simple model will also be the most feasible approach to be implemented as a behavioral control software of very limited micro-robots in the final physical implementation. We target a bottom-up approach in our model building as the final target system will be micro-robots, thus we need to model the desired “agency” also in our model. There are several ways to define “simplicity” for hypotheses, and thus also for their mathematical form of expression, which are mathematically formulated models (Sober, 2002). In our case, we aimed at a model that is capable of capturing the desired system dynamics and properties with a minimum amount of behavioral states, with a minimum amount of agent-internal memory (e.g., internal variables), with a minimum amount of global information available to the agents (e.g., gradient fields), and with a minimum amount of direct agent-to-agent communication.

In ethology, it is a long-known principle, that animal behaviors can be triggered reliably by physical stimuli that exceed a certain intensity, a phenomenon that is often called a “threshold” (Tinbergen, 1948; Lorenz, 1970). Such stimulus-response mechanisms are thus non-linear acting processes, which can lead to, especially in animal-to-animal interactions, to complex self-organization and self-regulation within social insect colonies (Bonabeau et al., 1999; Camazine et al., 2020; Lazic and Schmickl, 2021). It was found that such complex processes can be modeled well by multi-agent models in a bottom-up way if the mechanisms of agents employ threshold-based behavior triggers and modulators. The simplest types of such models have fixed stimulus-threshold mechanisms, these models have been for example developed for modeling ants (Bonabeau et al., 1996, 1998), honeybees (Schmickl et al., 2012; Szopek et al., 2021a) and termites (Feltell et al., 2008). Often, and also for our needs here, such fixed thresholds are not powerful enough to capture the observed dynamics in the system. Going beyond mere fixed threshold mechanism, also dynamic modulations of such threshold systems have been used, often called “threshold-reinforcement mechanism” (Theraulaz et al., 1998). Such types of models can capture processes like habituation and sensitization, two low-cognitive forms of learning (Castellucci et al., 1970). For example, a threshold-adaptation-based mechanism was successfully used to model the emergent degree of task specialization in ants with increasing colony size (Gautrais et al., 2002). In their study, Gautrais et al. have implemented the threshold-reinforcement mechanisms of their model in a way so that the workers of the colony “fixate” themselves more and more over time to specific tasks, thus turning from highly flexible generalists to specialists fixated on one or a few specific tasks. For our courtyard bee model, we seek the opposite outcome: We seek an as-simple-as-possible threshold-adaptation mechanism that captures the behavioral dynamics of courtyard bees, which alternate between periods in which they seek closeness to the queen and periods in which they stray out far away from the queen into the other regions of the hive. We interpret these periods as alternative phases of “habituation” and “dishabituation” (Castellucci et al., 1970) in combination with threshold-driven taxis-behavior in the temperature gradient field of the brood nest region and in the chemical pheromone gradient field originating from the queen. Such “on/off” dynamics of courtyard attendance have been reported by (Seeley, 1979) and are also reported from our own empirical observations in the study at hand.

While the model presented here is strikingly simple, it is still capable of producing dynamics of the queen agent and of the worker bee agents that qualitatively, and also quantitatively, resemble the observations we made in experiments with real bees. The model also captures well the honeybee queen court dynamics reported in literature. We incorporated an as-small-as-possible number of assumptions in our model, to honor the parsimony principle in our model building. However, our model still uses several parameters whose exact values are not known. These free parameters correspond to the properties of natural processes, such as the emission, diffusion and decay rates of the pheromones. Also, the parameters of our adaptation mechanisms (habituation, dishabituation) are not empirically derived, yet.

Another simplification is that we treat the queen pheromones as if they were transmitted exclusively by air. However, some important pheromones are cuticular carbohydrates and some of these substances are transferred by cleaning, licking, or other physical contact. Some other pheromones are footprint pheromones, deposited by walking on the comb. Since our model is a spatial model, this can be added in a future extension. It will require more studies to parameterize the model more reliably and to extend the model with mechanisms that are currently not implemented.

However, despite these simplifications, the model we describe here is able, as an emergent phenomenon by itself, to simulate the courtyard size on a honeycomb in a way that is statistically indistinguishable from empirical observations.

**FIGURE 11:**
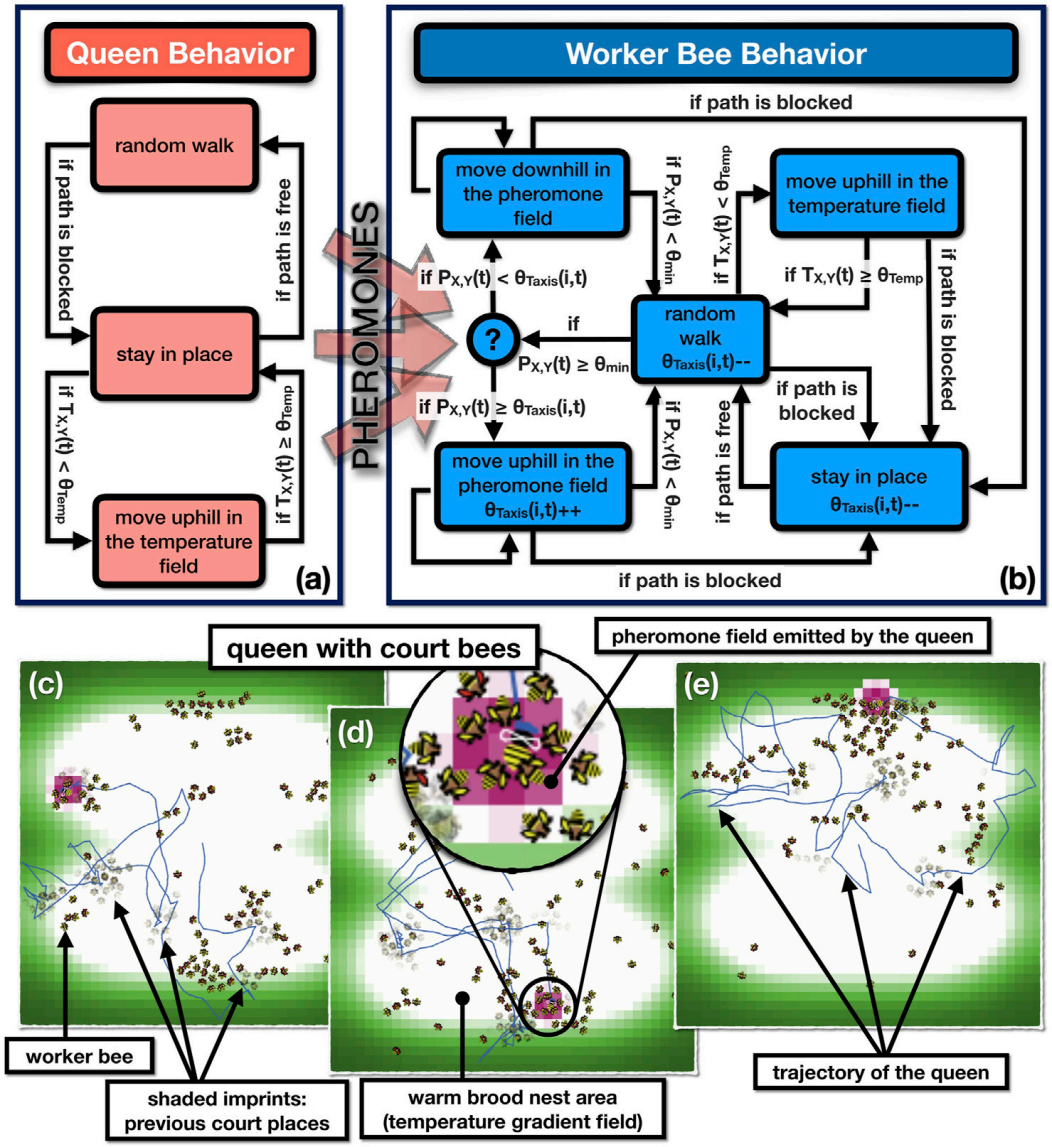
Agent-based model of the queen and the worker bees. Upper subfigures **(A,B)**: Basic computational structures in the agent-based model expressed as two interacting finite state machines. Colored boxes indicate behavioral states of agents, the arrows between the boxes indicate transitions between these states. At each arrow, the condition or the probability of the transition is given. Lower subfigures **(C-E)**: Screenshots depicting the basic emerging structures in the system: the pheromone field, the courts, the trajectories of the agents and the patio-temporal self-organization within the system.

This model is planned to be later used as a basis for generating the behavioral control of the robotic court bee surrogates. The core mechanics of this model are depicted in Figure 11. The top figures show the state machines that drive 1) the queen agent and 2) the worker bee agents. The lower graphs depict (c-e) exemplary simulation runs over a period of 50 h each. The emerging queen courts are depicted as follows: Each time step all worker bees stamp their body shape to the background of the simulation world with an opacity of 99%. Thus these “virtual body imprints” become only clearly visible if the worker bees stay for many time steps at the same place, like it is the case when they become court bees. The lower subfigures furthermore depict the queen’s trajectory over the simulated comb space and the thermal gradient that marks the brood nest area of the colony, where the queen usually resides. The thermal uphill behavior of the queen below a given temperature threshold models the queen’s locomotion behavior which prefers the warm brood nest area.

The presented model resembles the basic structures of a real beehive’s brood nest comb and is capable of reproducing the basic features of the activity patterns of its queen and worker bees. However, it is unclear how well it can generalize across different parameters of the hive, such as the number of worker bees or brood size, and environmental conditions, such as ambient temperature or humidity. To estimate the model parameters so that it aligns with such observations, one can use regression analysis to tune the model. Modeling the entire colony as a dynamic system with nested feedback loops would allow it to employ dynamic system identification techniques. These rely not only on observations, but with the suggested robotic system we will be able to actively perturb the system inputs in a controlled way to observe its response outside of the normal operating conditions.

**FIGURE 12:**
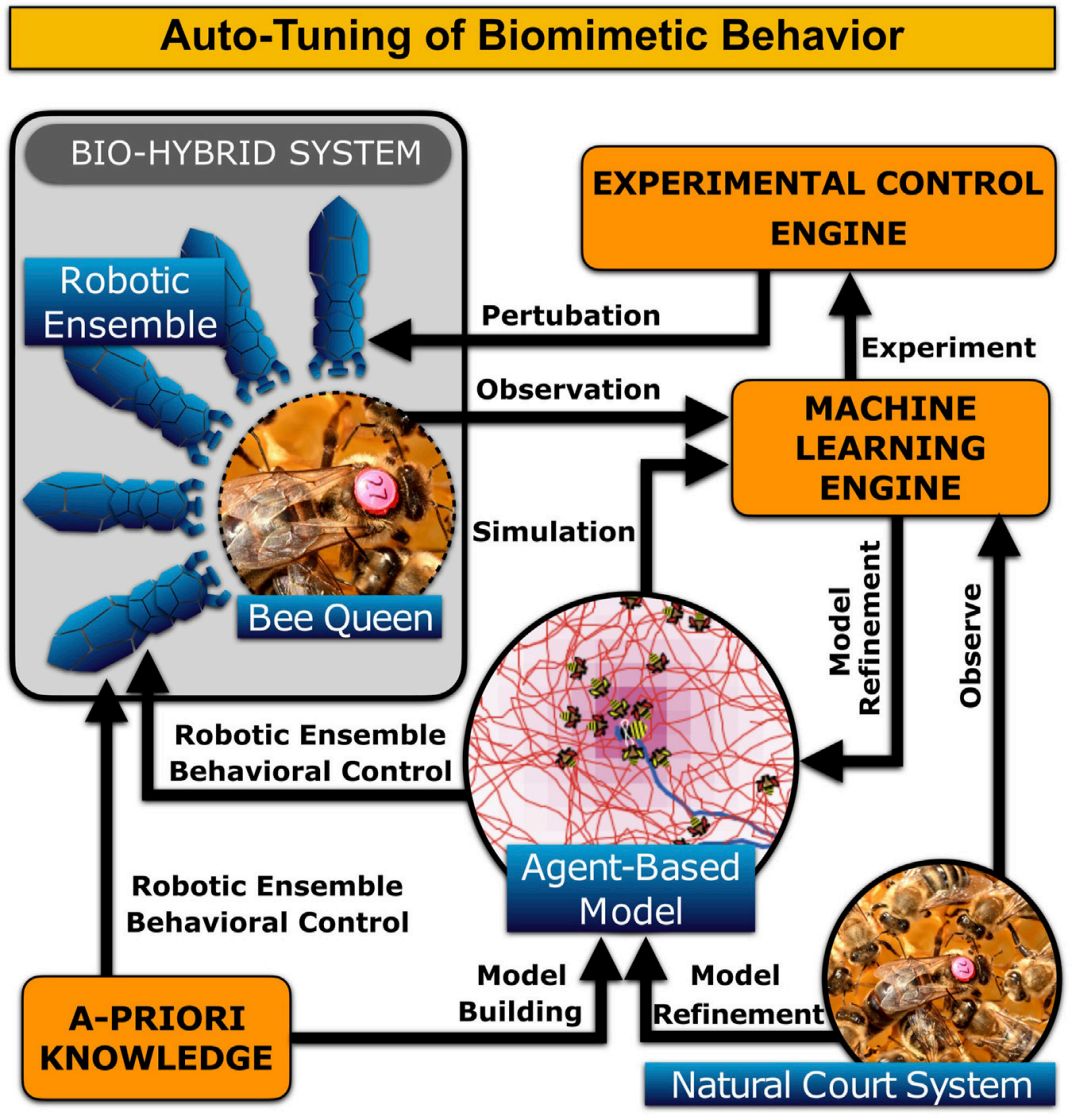
Overall concept of how the agent-based model of the queen court behavior can be distilled and refined *via* Machine Learning in combination with a set of auto-generated perturbation experiments.

In fact, direct control of the artificial court bees allows to decouple specific feedback loops. For the purposes of system identification, we can occasionally control the bee colony in an open loop manner. This will allow us to observe the colony’s response to abrupt changes in the court behavioral parameters, making an estimation of the model parameters easier, and would also identify structural deficiencies of the models used. Model identification could be performed in a hierarchical manner—once we identify the court-queen system, we can design perturbations to refine the court-queen-brood system, proceeding through the nested feedback loops as outlined in Figure 1. While the exact techniques to achieve optimal model identification are subject to speculation, drawing analogies from modern control theory and machine learning will provide us with tools that are more powerful than mere observations. Figure 12 shows a general schematic on our envisioned automated model refinement.

Such an automated model refinement and tuning will, in future, allow us to use the multi-agent model as a “driver algorithm” for the behavioral control of our planned physically embodied court-bee surrogates (biomimetic robots) to take over an active role in their colonies. These robots can for example alter the queen’s access to worker bees by regulating the frequencies at which these bees can physically access and feed her, while we can simultaneously observe the impact of this behavioral modulation on the court size and switching behavior dynamics. In parallel, we could reduce the number of workers approaching the queen by activating a fixed number of (robotic) court members at certain times. The natural workers would thus perceive fewer pheromones from the queen, which in turn would affect the dynamics of their switching behavior.

By also allowing the surrogates to feed the queen directly on-demand, we may boost her egg-laying activity, as this is associated with the feeding rate or feed the queen specific health-boosting or curing (medicinal) food without affecting the rest of the hive. It is well known that bees respond to increased brood activity by increasing their pollen foraging and thus pollination output (Fewell and Winston, 1992). In the approach presented here, we can take advantage of the fact that the queen bee is a central core unit in the hive, as she is the sole producer of brood in a healthy hive. The queen is a constantly pheromone releasing agent, modulating the worker bees’ behaviors via various feedbacks and threshold-based mechanisms with her unique pheromone bouquet, with its spatial spread and with its temporal dynamics (Seeley, 2009).

Besides these potential fields of application, basic research and a deeper fundamental understanding of the queen’s central role in colony integration and self-regulation is an important impact of the envisioned overall system. The introduction of robotic biomimetic surrogate agents allows for observing the colony over extended time periods, and to observe the system’s reaction to experimentally introduced perturbations of its equilibrium states. Processing the collected data by machine learning methods will allow us to construct high-fidelity models of the hive dynamics and its agent’s behaviors. These models will then serve to synthesize optimal control methods of the beehive through the biomimetic agents affecting the queen.

There is still a long way to go to stabilizing ecosystem intervention using a eusocial insect superorganism in combination with robots. However, along the way, much knowledge will be gained about the social interactions between the queen and the other individuals in the colony, opening the door to a better understanding of honeybee colony organization. Successfully (and positively) impacting pollination would prove the establishment of a biohybrid system between the robots and the honeybee queen (and by extension, the colony). Ultimately, the goal is to manage the pollination performance of the bee colony by controlling the protein requirement of the honeybee queen. In this way, the strength of plant pollination surrounding the colony can be controlled, making the biohybrid system a stabilizing element in the ecosystem.

## Data Availability

The raw data supporting the conclusion of this article will be made available by the authors, without undue reservation.

## Footnotes

1 Raspberry Pi Camera module 2 NoIR in 2019 and Raspberry Pi High Quality Camera with manually removed IR filter and 6 mm CS-mount lens in 2020 and 2021
