# Erratum: A minimally invasive approach towards “ecosystem hacking” with honeybees

**DOI:** 10.3389/frobt.2022.1058324

**Published:** 2022-11-02

**Authors:** Frontiers Production Office

**Affiliations:** Frontiers Media SA, Lausanne, Switzerland

**Keywords:** honeybees, micro-robotics, ecosystem hacking, swarm robotics, queen behavior

Due to a production error, there was an error in affiliation 1. Instead of “Artificial Life Lab, Institute of Biology, University of Graz, Prague, Austria”, it should be “Artificial Life Lab, Institute of Biology, University of Graz, Graz, Austria”.

The publisher apologizes for this mistake. The original version of this article has been updated.

